# Supplementary material for: MetaQTL: a package of new computational methods for the meta-analysis of QTL mapping experiments
Source: BMC Bioinformatics. 2007 Feb 8;8:49. doi: 10.1186/1471-2105-8-49 (PMC1808479; doi:10.1186/1471-2105-8-49)
Supplement: Additional File 7 — MetaQTL Package : jar file and tutorial. This Zip archive contains both the MetaQTL JAR file and the files of the tutorial. [file 1471-2105-8-49-S7.zip › org.inra.metaqtl/doc/org/metaqtl/graph/QtlTreeLayer.html]

QtlTreeLayer


|  |  |  |
| --- | --- | --- |
| **Overview** \| **Package** \| **Class** \| **Use** \| **Tree** \| **Deprecated** \| **Index** \| **Help** | |  |
| **PREV CLASS**   **NEXT CLASS** | **FRAMES**    **NO FRAMES**     **All Classes** |  |
| SUMMARY: NESTED \| FIELD \| CONSTR \| METHOD | DETAIL: FIELD \| CONSTR \| METHOD |  |


---


## org.metaqtl.graph Class QtlTreeLayer

```
java.lang.Object
  org.metaqtl.graph.AnchoredElement
      org.metaqtl.graph.Layer
          org.metaqtl.graph.QtlTreeLayer
```

---

``` public class QtlTreeLayer extends Layer ```

---

**Field Summary**

**Fields inherited from class org.metaqtl.graph.Layer**

`height, width`

**Fields inherited from class org.metaqtl.graph.AnchoredElement**

`x, y`


**Constructor Summary**

`QtlTreeLayer(double x,
double y)`


| **Method Summary** | |
| --- | --- |
| `void` | `attach(java.lang.Object object)`             Attach the given `object` to the layer. |
| `void` | `build(java.awt.Graphics2D graph)`             Build the layer. |
| `void` | `draw(java.awt.Graphics2D graph)`             Draws the layer on the given `graph` context. |
| `double` | `getYMax()` |
| `double` | `getYMin()` |
| `void` | `setChromAxe(ChromAxe axe)` |

**Methods inherited from class org.metaqtl.graph.Layer**

`getHeight, getWidth`

**Methods inherited from class org.metaqtl.graph.AnchoredElement**

`getX, getY, setX, setY`

**Methods inherited from class java.lang.Object**

`clone, equals, finalize, getClass, hashCode, notify, notifyAll, toString, wait, wait, wait`

**Constructor Detail**

### QtlTreeLayer

```
public QtlTreeLayer(double x,
                    double y)
```

**Parameters:**: `x` -: `y` -


**Method Detail**

### attach

```
public void attach(java.lang.Object object)
```

:   **Description copied from class: `Layer`**
:   Attach the given `object` to the layer.

    :   **Specified by:**: `attach` in class `Layer`
    :   **Parameters:**: `object` - the object to attach to the layer.

---


### build

```
public void build(java.awt.Graphics2D graph)
```

:   **Description copied from class: `Layer`**
:   Build the layer.

    :   **Specified by:**: `build` in class `Layer`

---


### draw

```
public void draw(java.awt.Graphics2D graph)
```

:   **Description copied from class: `Layer`**
:   Draws the layer on the given `graph` context.

    :   **Specified by:**: `draw` in class `Layer`

---


### getYMin

```
public double getYMin()
```

:   **Specified by:**: `getYMin` in class `Layer`

---


### getYMax

```
public double getYMax()
```

:   **Specified by:**: `getYMax` in class `Layer`

---


### setChromAxe

```
public void setChromAxe(ChromAxe axe)
```

:   **Parameters:**: `axe` -


---


|  |  |  |
| --- | --- | --- |
| **Overview** \| **Package** \| **Class** \| **Use** \| **Tree** \| **Deprecated** \| **Index** \| **Help** | |  |
| **PREV CLASS**   **NEXT CLASS** | **FRAMES**    **NO FRAMES**     **All Classes** |  |
| SUMMARY: NESTED \| FIELD \| CONSTR \| METHOD | DETAIL: FIELD \| CONSTR \| METHOD |  |


---
